# Eco-tourism, climate change, and environmental policies: empirical evidence from developing economies

**DOI:** 10.1057/s41599-023-01777-w

**Published:** 2023-05-31

**Authors:** Yunfeng Shang, Chunyu Bi, Xinyu Wei, Dayang Jiang, Farhad Taghizadeh-Hesary, Ehsan Rasoulinezhad

**Affiliations:** 1 School of Hospitality Administration, Zhejiang Yuexiu University, Zhejiang, China; 2 School of Economics, Tianjin University of Commerce, Tianjin, China; 3 School of Global Studies, Tokai University, Tokyo, Japan; 4 TOKAI Research Institute for Environment and Sustainability (TRIES), Tokai University, Tokyo, Japan; 5 Faculty of World Studies, University of Tehran, Tehran, Iran

**Keywords:** Environmental studies, Economics

## Abstract

Developing ecotourism services is a suitable solution to help developing countries improve the status of sustainable development indicators and protect their environment. The primary purpose of this paper is to find out the effects of green governance variables and carbon dioxide emissions on ecotourism for 40 developing economies from 2010 to 2021. The results confirmed a uni-directional causal relationship between the green governance indicator and the inflation rate of the ecotourism indicator. In addition, with a 1% improvement in the green governance index of developing countries, the ecotourism of these countries will increase by 0.43%. In comparison, with a 1% increase in the globalization index of these countries, ecotourism will increase by 0.32%. Moreover, ecotourism in developing countries is more sensitive to macroeconomic variables changes than in developed economies. Geopolitical risk is an influential factor in the developing process of ecotourism. The practical policies recommended by this research are developing the green financing market, establishing virtual tourism, granting green loans to small and medium enterprises, and government incentives to motivate active businesses.

## Introduction

**Fig. 1:**
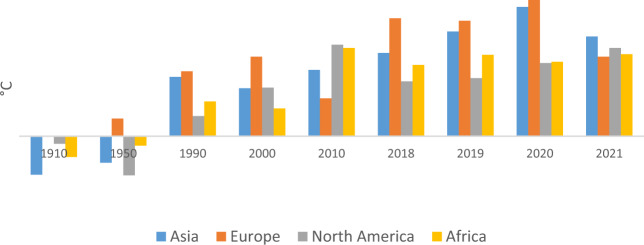
Surface temperature, °C, 1910–2021. Source: Authors from NOAA (https://www.ncei.noaa.gov/access/monitoring/climate-at-a-glance/global/time-series).

The challenge of climate change has become a primary threat to living on the Earth in the last centuries (Rasoulinzhad and Taghizadeh-Hesary, 2022). Many meetings of the countries at the regional and international level are held on the topics of environment and climate change. Regardless of environmental issues, population growth, and the lack of control of greenhouse gas emissions, industrialization has been the most crucial cause of the climate change crisis. Chao and Feng (2018) address human activity as the leading cause of climate change and express that this challenge is a potential threat to living on Earth. Woodward (2019) argued that climate change threats include the rise in global temperature, the melting of polar ice caps, and unprecedented disease outbreaks. Therefore, urgent policies and solutions are essential to control and lower the risk of global change. One of the signs of climate change is the increase in the average temperature of the Earth’s surface. Figure 1 shows the temperature data from 1910 to 2021 for the four continents of Asia, Europe, Africa, and North America.

The data in Fig. 1 shows that the air temperature has increased significantly over the past century, which has been more prominent in Asia and Europe. In 2021, we saw a decrease in temperature changes due to the spread of the Corona disease and a decrease in the rate of greenhouse gas emissions. However, the role of the Asian continent in increasing the global temperature has been more than other continents due to its large population and excessive consumption of fossil fuels.

During the past decades, the world’s countries have tried to formulate and implement various environmental policies collectively in the form of agreements or separately to fight environmental threats. Regarding international agreements, such things as the Paris Agreement of 2015, the Kyoto Protocol of 1997, the Montreal Protocol of 1987, and the Vienna Convention on the Protection of the Ozone Layer in 1985 can be addressed whose primary purpose is to integrate the goals and motivation of the international community to the world’s environmental threats. However, a group of earlier studies, such as Zheng et al. (2017), Takashima (2018), and Roelfsema et al. (2022), emphasized the inefficiency of these global agreements, especially after the left the USA from the Paris Agreement on 1 June 2017. The most important cause of this inefficiency has been the need for more motivation of countries to fulfill their international obligations towards environmental issues. However, many governments consider the threat of climate change only within their geographical boundaries and have tried to formulate and implement green policies to advance their environmental protection goals. These policies include green financial policies (green taxes, green subsidies), monetary policies (such as green loans and green financing), and cultural and social policies in line with sustainable development. The ultimate goal of these green policies is a green economy, an environmentally friendly economy, a zero carbon economy, or a sustainable economy. Lee et al. (2022) define the green economy as a broad concept comprising green industry, agriculture, and services. Centobelli et al. (2022) express that environmental sustainability should be more attention in the service sector owing to its penetration into social life and interactions.

Tourism and travel-related services are among countries’ main parts of the service sector. By creating the flow of tourists, tourism services can lead to capital transfer, job creation, cultural exchange (globalization), and increasing welfare in the country hosting the tours. According to the Yearbook of Tourism Statistics published by the World Tourism Organization, international tourism has increased from 522.2 billion US dollars in 1995 to nearly 1.86 trillion US dollars in 2019. This increase shows the importance of tourism services in generating income for countries, especially in the era of Corona and post-corona. Casado-Aranda et al. (2021) express that tourism services can be a central driver of economic growth recovery in post COVID era. Jeyacheya and Hampton (2022) argue that tourism can make high incomes for host countries leading to job creation and economic flourishing in destination cities for tourists.

An important issue mentioned in the corona era and relies on the post-corona era is the revitalizing of green economic growth. An important issue mentioned in the corona era and relying on the post-corona era is the revitalizing green economic growth (Bai et al., 2022; Werikhe, 2022), an opportunity that countries should pay more attention to in order to rebuild their economic activities. In other words, countries should plan their return to economic prosperity with environmental issues in mind. To this end, the issue of tourism finds a branch called Ecotourism or sustainable tourism which has environmental concerns and tries to help countries to improve environmental protection policies. *Ecotourism* is an approach based on environmental criteria, which is opposed to over-tourism (a type of tourism that disrupts the protection of the environment and destroys natural resources). The International Ecotourism Society defines *Ecotourism* as an efficient way to conserve the environment and improve local people’s well-being. It can be said that Ecotourism, along with various economic advantages (income generation, job creation, globalization, poverty alleviation), will bring environmental protection to the world’s countries, achieving the goals of green economic growth recovery and sustainable development. Xu et al. (2022) consider Ecotourism as one of the essential components of achieving sustainable development in the post-corona era.

Ecotourism in developing countries has more priorities compared to developed economies. Firstly, developing countries are often countries with financial problems of the government, and the governments in these countries need more capital to advance sustainable development goals. Therefore, developing ecotourism services can be a suitable solution to help these countries improve the status of sustainable development indicators and protect their environment. Second, due to the spread of the Corona disease, developing countries have experienced numerous bankruptcy in the tourism services sector. Therefore, promoting ecotourism in these countries is of great importance in the post-corona era. Third, developing countries have a high share in the emission of greenhouse gases in the world due to their high dependence on fossil fuels and the lack of advanced green technologies. Fourth, due to bureaucratic processes, high cost, and lack of market transparency, greenwashing may happen in developing economies’ ecotourism industry, meaning that a company serving ecotourism services makes its activities seem more sustainable and ethical than they are. The term “greenwashing” can harshly impact the future development path of the ecotourism industry in developing economies. According to the reasons mentioned above, developing ecotourism in developing countries can be an essential factor in controlling and reducing greenhouse gas emissions in these countries.

This paper tries to contribute to the existing literature from the following aspects:

1. Calculating the ecotourism index for selected countries based on the criteria for measuring sustainable tourism stated by the World Tourism Organization in the United Nations. Considering that there is no specific index for ecotourism, the calculation of ecotourism in this article will be innovative.
2. Measuring the green governance index as a proxy for environmental policies for selected countries based on the Environment Social and Governance (ESG) data.
3. Selecting a sample of 40 developing countries from different geographical regions to calculate the interconnections between ecotourism, green governance, and climate change
4. Making a further discussion to address the role of uncertainty and the developing level of countries in the relationship between ecotourism and explanatory variables.

The main results confirm the existence of a uni-directional causal relationship running from the green governance indicator and inflation rate to the ecotourism indicator. In addition, with a 1% improvement in the green governance index of developing countries, the ecotourism of these countries will increase by 0.43%. A 1% increase in the globalization index of these countries accelerates ecotourism by 0.32%.

Moreover, ecotourism in developing countries is more sensitive to macroeconomic variables changes than in developed economies. Geopolitical risk is an influential factor in the developing process of ecotourism. The practical policies recommended by this research are developing the green financing market, establishing virtual tourism, granting green loans to small and medium enterprises, and government incentives to motivate active businesses.

The paper in continue is organized as follows: section “Literature review” provides a short literature review to determine the gaps this research seeks to fill. Section “Data and model specification” argues data and model specification. The following section represents empirical results. Section “Discussion” expresses discussion, whereas the last section provides conclusions, policy implications, research limitations, and recommendations to research further.

## Literature review

This part of the article analyzes and classifies the previous literature on ecotourism and sustainable development in a rational and structured way. The importance of tourism in economic growth and development has been discussed in previous studies. However, the study of the effect of tourism on climate change has received little attention. Especially the relationship between sustainable tourism, climate change, and environmental policies is a problem that has yet to receive the attention of academic experts.

A group of previous studies has focused on the place of tourism in economic development and growth. Holzner (2011) focused on the consequences of tourism development on the economic performance of 134 countries from 1970 to 2007. They found out that excessive dependence on tourism income leads to Dutch disease in the economy, and other economic sectors need to develop to the extent of the tourism sector. In another study, Sokhanvar et al. (2018) investigated the causal link between tourism and economic growth in emerging economies from 1995 to 2014. The main results confirmed that the linkage is country-dependent. Brida et al. (2020) studied 80 economies from 1995 to 2016 to determine how tourism and economic development are related. The paper’s conclusions highlighted tourism’s-positive role in economic activities.

Another group of previous studies has linked tourism to sustainability targets. Sorensen and Grindsted (2021) expressed that nature tourism development has a positive and direct impact on achieving sustainable development goals of countries. In a new study, Li et al. (2022) studied the impacts of tourism development on life quality (as one of the sustainable development goals defined by the UN in 2015) in the case of Japan. They found that tourism development positively impacts the quality of life of age groups in the country. Ahmad et al. (2022) explored the role of tourism in the sustainability of G7 economies from 2000–2019. The primary findings revealed the positive impact of tourism arrivals on sustainable economic development. Zekan et al. (2022) investigated the impact of tourism on regional sustainability in Europe. They concluded that tourism development increases transport, leading to increased carbon dioxide emissions. Therefore, tourism development causes environmental pollution.

Tourism that can pay attention to environmental issues is called “ecotourism.” Many new studies have studied different dimensions of ecotourism. Lu et al. (2021) expanded the concept of the ecotourism industry. The significant results expressed that smart tourist cities are essential for efficient ecotourism in countries. Thompson (2022) expressed the characteristics of ecotourism development through survey methodology. The results confirmed the importance of transparent regulations, government support, and social intention to promote ecotourism. In another study, Heshmati et al. (2022) employed the SWOT analysis method to explore the critical success factors of ecotourism development in Iran. They found that legal documentation and private participation are major influential factors in promoting ecotourism in Iran. In line with the previous research, Hosseini et al. (2021) tried to explore the influential factors in promoting ecotourism in Iran by employing a SWOT analysis. They depicted that attracting investors is essential to enhance ecotourism projects in Iran. Hasana et al. (2022) reviewed research to analyze the earlier studies about ecotourism. The conclusions expressed that ecotourism is necessary for environmental protection. However, it is a challenging plan for the government, and they should carry out various policies toward ecotourism development. Kunjuraman et al. (2022) studied the role of ecotourism on rural community development in Malaysia. The significant results confirmed that ecotourism could transfer-positive impacts.

Several earlier studies have concentrated on the characteristics of ecotourism in different developed and developing economies. For example, Ruhanen (2019) investigated the ecotourism status in Australia. The paper concluded that the country could potentially make a larger share of ecotourism to the entire local tourism industry. Jin et al. (2022) studied the role of local community power on green tourism in Japan. They concluded that the concept of agricultural village activity and regional support positively influences the development of green tourism in Japan as a developed economy. Choi et al. (2022) sought to find aspects of ecotourism development in South Korea. The preliminary results confirmed the importance of green governance and efficient regulation to promote a sustainable tourism industry. Baloch et al. (2022) explored the ecotourism specifications in the developing economy of Pakistan. They found that Pakistan’s ecotourism needs government support and the social well-being of the visited cities. Sun et al. (2022) studied ecotourism in China. They concluded that there is imbalanced development of ecotourism among Chinese provinces due to the need for more capital to invest in all ecotourism projects throughout the Chinese cities. Tajer and Demir (2022) analyzed the ecotourism strategy in Iran. They concluded that despite various potentials in the country, insufficient capital, lack of social awareness, and political tension are the major obstacles to promoting a sustainable tourism industry in Iran.

Another group of earlier studies has drawn attention to promoting eco-tourism in the post COVID era. They believe that the corona disease has created an excellent opportunity to pay more attention to environmental issues and that countries should move towards sustainable development concepts such as sustainable (eco) tourism in the post-corona era. Soliku et al. (2021) studied eco-tourism in Ghana during the pandemic. The findings depicted the vague impacts of a pandemic on eco-tourism. Despite the short-term negative consequence of the pandemic on eco-tourism, it provides various opportunities for developing this sector in Ghana. Hosseini et al. (2021) employed the Fuzzy Dematel technique to find solutions for promoting eco-tourism during COVID-19. They found out that planning to increase the capacity of eco-tourism and incentive policies by governments can help promote the eco-tourism aspect under the pandemic’s consequences. Abedin et al. (2022) studied the consequence of COVID-19 on coastal eco-tourism development. The primary findings confirmed the negative impacts of a pandemic on the development of eco-tourism.

A review of previous studies shows that tourism can positively impact green growth and sustainable development. Sustainable tourism can be used as a policy to deal with the threat of climate change. This issue needs more attention in the corona and post-corona eras. Because in the post-corona era, many countries have sought to revive green economic growth, and ecotourism can be one of the tools to achieve it. As observed, a detailed study of the relationship between climate change, ecotourism, and environmental policies has yet to be done. Therefore, this research will address and fill this literature gap.

## Data and model specification

### Data description

The paper seeks to find the relationship between climate change, ecotourism, and environmental policy for the panel of 40 developing economies from different regions from 2010 to 2021 (480 observations). The sample size could have been more extensive due to the lack of information on some variables. However, there are 480 observations in the data analysis of the data panel; therefore, the number of samples selected is acceptable.

To determine the proxies for main variables, CO2 emissions per capita are selected as the proxy for climate change. Many earlier studies (e.g., Espoir et al., 2022) have employed this variable as an appropriate variable representing the status of climate change. Regarding ecotourism, the World Tourism Organization proposed some measurements of sustainable tourism, and also following Yusef et al. (2014), the entropy weight method is employed to calculate a multi-dimensional ecotourism indicator comprising per capita green park area (square meters), gross domestic tourism revenue (US dollars), the ratio of good air quality (%), green transport, renewable water resources (km3) and deforestation rate (%). It is a novel ecotourism indicator that can show the ecotourism status in countries.

In addition, the green governance index is calculated as a proxy for environmental policy. Principally, the Environment, Social, and Governance (ESG) data from World Bank are gathered to calculate this variable. With the improvement of the Green Governance Index, the quality of environmental policies will also increase, and vice versa. With the adverseness of the Green Governance Index, the efficiency of environmental policies will decrease.

Regarding control variables, the inflation rate as an influential factor in tourism flows is selected. The importance of this variable to promoting/declining tourism flows has been drawn to attention by some earlier studies, such as Liu et al. (2022). The inflation rate can raise the total cost of travel, causing a reduction in tourism flows, while any reduction in the inflation rate can increase the intention of tourists to travel. In addition, the KOF globalization index provided by the KOF Swiss Economic Institute is another control variable. A country with a higher degree of globalization means more readiness to accept tourists from countries with different cultures and religions.

### Model specification

According to the variables mentioned above, 40 examined developing countries from 2010 to 2021, the panel co-integration model can be written as Eq. 1:

1 $$ETOR_{i,t} = \alpha _0 + \beta _1CO2_{i,t} + \beta _2GGI_{i,t} + \beta _3INF_{i,t} + \beta _4GLOB_{i,t} + e_{i,t}$$

ETOR indicates the ecotourism index, while CO2, GGI, INF, and GLOB denote Carbon dioxide emissions per capita, green governance index, inflation rate, and globalization index, respectively. *i* is 1,2,…,40 and shows examined developing economies, while t is time and contains 2010, 2011,..,2021.

Prior to the estimation of coefficients of Eq. 1, the panel unit root tests are employed to find out whether the series is stationary. To this end, three tests of LLC (Levin et al., 2002), Breitung’s test (2000), and the PP-Fisher test (Philips and Perron, 1988). If all the variables are stationary at the first level of difference (I(1)), a panel co-integration test can be conducted to explore whether the model is spurious. To this end, Kao’s co-integration test (1999) and Pedroni’s residual co-integration test (2004) are conducted. If the co-integration relationship exists among variables, the panel causality test can be run to determine the causal linkages among variables. In this paper, the two steps of Engle and Granger (1987)‘s test, which is based on the error correction model (ECM) is used as Eqs. 2–6:

2 $$\begin{array}{l}\Delta ETOR_{i,t} = \theta _{1,i} + \mathop {\sum}\nolimits_{k = 1}^m {\theta _{1.1.i,k}} \Delta ETOR_{i,t - k} + \mathop {\sum}\nolimits_{k = 1}^m {\theta _{1.2.i,k}} \\ \Delta CO2_{i,t - k} + \mathop {\sum}\nolimits_{k = 1}^m {\theta _{1.3.i,k}} \Delta GGI_{i,t - k} + \mathop {\sum}\nolimits_{k = 1}^m {\theta _{1.4,i,k}} \Delta INF_{i,t - k}\\\qquad\qquad\;\;\, + \mathop {\sum}\nolimits_{k = 1}^m {\theta _{1.5.i,k}} \Delta GLOB_{i,t - k} + \lambda _{1,i}ECT_{i,t - 1} + \mu _{1,i,t}\end{array}$$

3 $$\begin{array}{l}\Delta CO2_{i,t} = \theta _{2,i} + \mathop {\sum}\nolimits_{k = 1}^m {\theta _{2.1.i,k}} \Delta ETOR_{i,t - k} + \mathop {\sum}\nolimits_{k = 1}^m {\theta _{2.2.i,k}} \\ \Delta CO2_{i,t - k} + \mathop {\sum}\nolimits_{k = 1}^m {\theta _{2.3.i,k}} \Delta GGI_{i,t - k} + \mathop {\sum}\nolimits_{k = 1}^m {\theta _{2.4,i,k}} \Delta INF_{i,t - k}\\\qquad\qquad\;\;\, + \mathop {\sum}\nolimits_{k = 1}^m {\theta _{2.5.i,k}} \Delta GLOB_{i,t - k} + \lambda _{2,i}ECT_{i,t - 1} + \mu _{2,i,t}\end{array}$$

4 $$\begin{array}{l}\Delta GGI_{i,t} = \theta _{3,i} + \mathop {\sum}\nolimits_{k = 1}^m {\theta _{3.1.i,k}} \Delta ETOR_{i,t - k} + \mathop {\sum}\nolimits_{k = 1}^m {\theta _{3.2.i,k}} \\ \Delta CO2_{i,t - k} + \mathop {\sum}\nolimits_{k = 1}^m {\theta _{3.3.i,k}} \Delta GGI_{i,t - k} + \mathop {\sum}\nolimits_{k = 1}^m {\theta _{3.4,i,k}} \Delta INF_{i,t - k}\\\qquad\qquad\;\;\, + \mathop {\sum}\nolimits_{k = 1}^m {\theta _{3.5.i,k}} \Delta GLOB_{i,t - k} + \lambda _{3,i}ECT_{i,t - 1} + \mu _{3,i,t}\end{array}$$

5 $$\begin{array}{l}\Delta INF_{i,t} = \theta _{4,i} + \mathop {\sum}\nolimits_{k = 1}^m {\theta _{4,1.i,k}} \Delta ETOR_{i,t - k} + \mathop {\sum}\nolimits_{k = 1}^m {\theta _{4.2.i,k}} \\ \Delta CO2_{i,t - k} + \mathop {\sum}\nolimits_{k = 1}^m {\theta _{4.3.i,k}} \Delta GGI_{i,t - k} + \mathop {\sum}\nolimits_{k = 1}^m {\theta _{4.4,i,k}} \Delta INF_{i,t - k}\\\qquad\qquad\;\;\, + \mathop {\sum}\nolimits_{k = 1}^m {\theta _{4.5.i,k}} \Delta GLOB_{i,t - k} + \lambda _{4,i}ECT_{i,t - 1} + \mu _{4,i,t}\end{array}$$

6 $$\begin{array}{l}\Delta GLOB_{i,t} = \theta _{4,i} + \mathop {\sum}\nolimits_{k = 1}^m {\theta _{4.1.i,k}} \Delta ETOR_{i,t - k} + \mathop {\sum}\nolimits_{k = 1}^m {\theta _{4.2.i,k}} \\ \Delta CO2_{i,t - k} + \mathop {\sum}\nolimits_{k = 1}^m {\theta _{4.3.i,k}} \Delta GGI_{i,t - k} + \mathop {\sum}\nolimits_{k = 1}^m {\theta _{4.4,i,k}} \Delta INF_{i,t - k}\\\qquad\qquad\;\;\, + \mathop {\sum}\nolimits_{k = 1}^m {\theta _{4.5.i,k}} \Delta GLOB_{i,t - k} + \lambda _{4,i}ECT_{i,t - 1} + \mu _{4,i,t}\end{array}$$

In the above Equations, Δ is the first differences of variables, while *θ* and ECT represent the fixed country effect and error correction term.

The next step is the long-run panel co-integration estimations. To this end, Fully Modified OLS (FMOLS) and Dynamic OLS (DOLS) as robustness checks are conducted, which are two famous panel co-integration estimators (Rasoulinezhad, 2018). The FMOLS estimator has various advantages. It allows serial correlation, endogeneity, and cross-sectional heterogeneity (Erdal and Erdal, 2020).

## Empirical results

In this section, we will implement the experimental research model. The purpose of implementing an econometric model based on panel data is to find the effects of green governance variables and carbon dioxide emissions on ecotourism. As the first step, the panel unit root tests are conducted. The results are reported in Table 1 as follows:

**Table 1:** Panel unit root test findings.

Test	*ETOR*	*CO2*	*GGI*	*INF*	*GLOB*
LLC:					
Level	−4.53 (0.130)	−2.49 (0.72)	−0.50 (0.35)	−4.33 (0.57)	−3.30 (0.94)
*D*()	−14.39 (0.00)	−13.93 (0.00)	−18.44 (0.00)	−23.59 (0.00)	−8.49 (0.00)
Breitung:					
Level	0.184 (0.47)	3.14 (0.10)	0.10 (0.55)	0.12 (0.73)	0.175 (0.26)
*D*()	−6.49 (0.00)	−9.13 (0.00)	−10.11 (0.00)	−7.04 (0.00)	−7.15 (0.00)
PP-Fisher:					
Level	13.43 (0.54)	37.50 (0.34)	43.05 (0.03)	31.95 (0.20)	22.60 (0.41)
*D*()	323.31 (0.00)	210.49 (0.00)	565.10 (0.00)	498.05 (0.00)	344.94 (0.00)

*ETOR*, *CO2*, *GGI*, *INF*, and *GLOB* are eco-tourism indicator, carbon dioxide emissions per capita, green governance indicator, inflation rate and globalization index, respectively.

Source: Authors.

According to Table 1, all three-panel unit root tests depict that all series are non-stationary at the level and become stationary after a first difference. Next, the panel co-integration tests are conducted, and their results are represented in Tables 2 and 3:

**Table 2:** Panel co-integration test (Pedroni technique).

	*t*-stat	*p*-value
Panel *v*-stat	0.249	0.019
Panel *r*-stat	0.042	0.009
Panel PP-stat	−2.74	0.003
Panel ADF-stat	−0.60	0.392
Group *r*-stat	−0.492	0.030
Group PP-stat	−3.502	0.000
Group ADF-stat	0.001	0.287

Source: Authors.

**Table 3:** Panel co-integration test (Kao technique).

	*t*-stat	*p*-value
ADF	−6.13	0.003

Source: Authors.

The two-panel co-integration tests’ findings confirm the presence of co-integration linkages among variables.

The panel causality test studies the short-term and long-term causal relationship among variables. Table 4 reports the results of the panel causality check as follows:

**Table 4:** Panel causality test.

Dependent variable	Explanatory variables					
	Short-term					Long-term
	Δ*ETOR*	Δ*CO2*	Δ*GGI*	Δ*INF*	Δ*GLOB*	ECT
Δ*ETOR*	–	−0.239 (0.00)	0.501 (0.042)	−0.113 (0.031)	0.429 (0.533)	9.832 (0.00)
Δ*CO2*	−0.39 (0.03)	–	−0.392 (0.00)	0.192 (0.443)	0.103 (0.23)	0.403 (0.45)
Δ*GGI*	0.45 (0.594)	0.223 (0.49)	–	−0.23 (0.001)	0.553 (0.684)	2.845 (0.013)
Δ*INF*	0.32 (0.119)	0.342 (0.64)	−0.21 (0.00)	–	−0.32 (0.053)	10.449 (0.32)
Δ*GLOB*	0.13 (0.023)	0.943 (0.32)	0.192 (0.04)	−0.13 (0.024)	–	6.443 (0.075)

*ETOR*, *CO2*, *GGI*, *INF*, and *GLOB* are eco-tourism indicator, carbon dioxide emissions per capita, green governance indicator, inflation rate and globalization index, respectively.

Source: Authors.

According to Table 4, there is a uni-directional causal relationship between the green governance indicator and the inflation rate of the ecotourism indicator. At the same time, there is a bi-directional causal relationship between carbon dioxide emissions and ecotourism indicators, confirming the existence of the feedback effect. In addition, there is only short-term causality from the green governance indicator to carbon dioxide emissions. In contrast, ecotourism and the globalization index have a uni-directional causal linkage. In the short term, improving ecotourism can cause globalization and reduce carbon emissions in developing economies. Regarding the long-term causality, it can be concluded that the ECT of ecotourism, green governance index, and globalization index are statistically significant. These three variables are major adjustment variables when the system departs from equilibrium.

In the last stage, the long-run estimations are done through FMOLS and DOLS estimators. Table 5 lists the results of the estimations by these two-panel co-integration estimators:

**Table 5:** FMOLS and DOLS estimations.

Explanatory variable	FMOLS	DOLS (Robustness check)
*GGI*	0.432 (0.003)	0.102 (0.023)
*CO2*	−0.231 (0.394)	−0.001 (0.511)
*INF*	−0.344(0.000)	−0.229 (0.003)
*GLOB*	0.328 (0.023)	0.492 (0.007)

*CO2, GGI, INF*, and *GLOB* are carbon dioxide emissions per capita, green governance indicator, inflation rate and globalization index, respectively.

Source: Authors.

Based on FMOLS estimation, it can be concluded that the Green Governance index has a positive and significant coefficient in such a way that with a 1% improvement in the green governance index of developing countries, the ecotourism of these countries will increase by 0.43%. By improving the state of green governance, the quality of formulated and implemented green policies in these countries will increase, improving the conditions of ecotourism development. This finding aligns with Agrawal et al. (2022) and Debbarma and Choi (2022), who believe that green governance is essential to sustainable development. In the case of carbon dioxide emissions, the coefficient of this variable is not statistically significant. In other words, the variable of carbon dioxide emissions per capita has no significant effect on ecotourism in developing countries. The inflation rate has a significant negative effect on ecotourism. With a 1% increase in the general prices of goods and services in developing countries, ecotourism will decrease by 0.34%. This finding aligns with Rahman (2022), who showed a negative relationship between inflation and sustainable development in their research. An increase in inflation means an increase in the total cost of a tourist’s trip to the destination country, inhibiting the growth of tourist services.

Regarding the globalization variable, this variable has a significant positive effect on the ecotourism of developing countries. With a 1% increase in the globalization index of these countries, ecotourism will increase by 0.32%. Globalization means more interaction with the world’s countries, acceptance of different cultures and customs, more language learning in society, more acceptance of tourism, and development of tourist services in the country. This finding is consistent with the results of Akadiri et al. (2019), who confirmed that globalization is one of the crucial components in tourism development.

The DOLS estimator was also used to ensure the obtained findings’ validity. The results of this method are shown in Table 5. The signs of the coefficients are consistent with the results obtained by the FMOLS method. Therefore, the validity and reliability of the obtained coefficients are confirmed.

## Discussion

In this section, we will briefly discuss the relationship between ecotourism and climate change and the environmental policy considering the uncertainty and the relationship between variables in developed and developing countries.

### Consideration of uncertainty

Uncertainty as a primary reason for risk has become a research issue in recent decades. Uncertainty can make the future unpredictable and uncontrollable, affecting economic decision-making. Regarding tourism, the impacts of uncertainty have been drawn to attention by several earlier studies (e.g., Dutta et al., 2020; Das et al., 2020; and Balli et al., 2019; Balli et al., 2018). In general, uncertainty in the tourism industry reflects tourists’ concerns and consumption habits in the way that by increasing uncertainty, it is expected that tourists make sense of risks and postpone their tourism activities, and vice versa; in the sphere of certainties, the various risks are clear, and tourists can make rational decisions for their tourism plans and activities. In order to explore the impacts of uncertainties on eco-tourism of the examined developing economies, the geopolitical risk index (GPR) as a proxy for economic policy uncertainty index is gathered and added as a control variable to Eq. 1. The estimations results by FMOLS are reported in Table 6 as follows.

**Table 6:** FMOLS estimation by considering uncertainty variable.

Explanatory variable	Coefficient	*p*-value
*GGI*	0.039	0.009
*CO2*	−0.110	0.583
*INF*	−0.113	0.048
*GLOB*	0.018	0.001
*GPR*	−0.692	0.058

*CO2, GGI, INF, GPR,* and *GLOB* are carbon dioxide emissions per capita, green governance indicator, inflation rate, geopolitical risk index and globalization index, respectively.

Source: Authors.

According to Table 6, the uncertainty (geopolitical risk) has a negative coefficient meaning that with a 1% increase in geopolitical risk, the eco-tourism industry in the examined developing countries decreases by approximately 0.69%. The signs of coefficients of other variables align with the earlier findings, represented in Table 5. In addition, the magnitude of the impact of geopolitical risk is larger than the impacts of other variables highlighting the importance of lower geopolitical risk in these economies to reach sustainable tourism targets.

### Difference in developed and developing economies

Considering the different structures and financial power of these two groups of countries, the relationship between the variables mentioned in these two groups is expected to be different. In the previous section, the results for the group of developing countries showed that the Green Governance index has a positive and significant coefficient. In the case of carbon dioxide emissions, the coefficient of this variable is not statistically significant. The inflation rate has a significant negative effect on ecotourism. Regarding the globalization variable, it can be mentioned that this variable has a significant positive effect on the ecotourism of developing countries. In order to analyze the relationship between variables in the developed countries, the top 10 countries with the highest HDI in 2021 are selected (Switzerland (0.962), Norway (0.961), Iceland (0.959), Hong Kong (0.952), Australia (0.951), Denmark (0.948), Sweden (0.947) and Ireland (0.945)). The selected variables, explained in section “Data and model specification”, are collected from 2010 to 2021. The panel unit root tests confirmed that all series are non-stationary at the level and become stationary after a first difference. In addition, the presence of co-integration linkages among variables is revealed by the panel co-integration test. The panel co-integration estimator of FMOLS is employed to study the long-term relationship among variables. The findings are reported in Table 7 as follows:

**Table 7:** FMOLS estimation for developed panel of countries.

Explanatory variable	Coefficient	*p*-value
*GGI*	0.238	0.0442
*CO2*	−0.034	0.0039
*INF*	−0.039	0.0145
*GLOB*	0.0139	0.0674

*CO2, GGI, INF*, and *GLOB* are carbon dioxide emissions per capita, green governance indicator, inflation rate and globalization index, respectively.

Source: Authors.

According to the estimated coefficients, the green governance indicator positively and statistically significantly impacts ecotourism in the examined developed economies. However, the magnitude of the impact of this variable is more considerable for developing countries because these countries have more imbalances in markets and regulations. Therefore, the presence of good green tourism can have a more positive effect on advancing the goal of ecotourism. Contrary to the findings of developing countries, carbon dioxide emission in developed countries has a negative and significant effect, meaning that with an increase of 1% in carbon dioxide in developed countries, the level of ecotourism becomes more unfavorable by 0.034%. Moreover, inflation and globalization variables have significant negative and positive coefficients, respectively. However, the magnitudes of these two variables’ coefficients are also higher in developing countries. Ecotourism in developing countries is more sensitive to changes in macroeconomic variables such as green governance, globalization, and inflation.

Another difference between eco-tourism in developed and developing economies may be interpreted through the term “greenwashing,” introduced by Westerveld in 1986 (Maichum et al., 2016). In developing countries, due to the economic structure, limited knowledge, bureaucratic process, lack of legal eco-certification, and imperfect competition, a company involved in the eco-tourism industry makes an unsubstantiated claim to deceive consumers into accepting the company’s services are in line with environmental protection policies. Hence, green governance in developing countries should have another role in regulating the eco-tourism market to lower the threat of greenwashing in eco-tourism services.

## Conclusions and policy recommendations

### Concluding remarks

The findings of econometric modeling revealed the relationship between environmental policies, climate change, and ecotourism. Based on the findings of the econometric model, the following conclusions can be presented:

1. A uni-directional causal relationship runs from the green governance indicator and inflation rate to the ecotourism indicator, which means that any changes in green governance and inflation rate cause changes in ecotourism, which is vital for developing economies where governance and inflation rate are two crucial issues.
2. There is a bi-directional causal relationship between carbon dioxide emissions and ecotourism indicators, confirming the existence of the feedback hypothesis, expressing that in developing economies, any policies related to ecotourism cause changes in CO2 emissions and vice versa.
3. There is only short-term causality from the green governance indicator to carbon dioxide emissions, whereas there is a uni-directional causal linkage from ecotourism to the globalization index. In other words, in the short term, improving ecotourism can cause globalization and reduce carbon emissions in developing economies.
4. By improving green governance in developing economies, the quality of formulated and implemented green policies in these countries will increase, improving the conditions of ecotourism development.
5. An increase in the inflation rate raises the total cost of a tourist’s trip to developing economies, inhibiting the growth of eco-tourist services.
6. Globalization means more interaction with the world’s countries, acceptance of different cultures and customs, more language learning in society, more acceptance of tourism, and development of tourist services in developing countries.

### Policy implications

In order to achieve the promotion of ecotourism in developing countries, the implementation of integrated and effective strategic and practical policies is of great importance. According to the concluding remarks mentioned, practical policies are presented as follows for enhancing ecotourism in developed countries. The development of ecotourism requires the improvement of various infrastructures and mechanisms, which depends on the implementation of projects related to ecotourism in developing countries. Because most countries do not have enough financial power to invest in such projects, developing the *green financing market* can be one of the critical practical solutions. The green financing tool can increase the investment risk and return on investment in such projects, and as a result, the participation of the private sector in these projects will increase. With information and communication technology development, *virtual tourism* can solve many environmental issues related to human physical presence. Virtual tourism is one of the branches of tourism services that provide people with destinations, places of interest, and tourist attractions with full quality but in virtual form. Another practical policy is granting *green loans* to small and medium enterprises active in ecotourism. Despite the organizational agility, these companies do not have the significant financial power to develop different sectors of ecotourism; therefore, the cooperation of the banking industry of developing countries by providing green loans (with low-interest rates) can motivate small and medium-sized companies in the field of activities related to ecotourism. *Government incentives* to motivate businesses active in ecotourism and *government deterrent policies* (green tax) from businesses active in the field of tourism to lead them to increase the share of ecotourism in their activities can be a proper operational strategy. In developing countries, the role of government and green governance is vital in advancing the goals of ecotourism. By improving the level of its green governance, the government can create efficient policies, regulations, and social tools to create motivation and desire to accept ecotourism, an essential and undeniable issue in developing societies. Creating a *guarantee fund* for ecotourism companies in developing countries is another practical policy to support these companies financially. Guarantee funds can be established with the participation of the people of ecotourism destinations in order to strengthen the financial strength of ecotourism companies in these destinations.

### Limitations and recommendations to further research

This research had a practical and innovative contribution to the literature on ecotourism in developing countries. The findings obtained from the econometric model analysis provided appropriate practical and strategic policies to the policymakers of countries interested in the development of ecotourism. However, access to data related to the ecotourism index and sustainable development of developing countries due to the lack of community in a specific database is considered one of the critical limitations of this research. This limitation caused many developing countries to be excluded from the research sample, which may have created a deviation in the research. Adding more countries to the test sample in future research is suggested to obtain complete and accurate results. Also, due to the outbreak of the Corona pandemic at the end of 2019 and the Russia-Ukraine war since the beginning of 2022, it is suggested that these two variables be included in the econometric model as an illusion in order to analyze their effects on the ecotourism of the countries of the world. Using other econometric methods, such as artificial neural networks, is suggested to model ecotourism in different countries. Complex modeling by taking into account trends and trends to predict the relationship between variables in the future will be an essential step in formulating effective programs in ecotourism.

## Data Availability

The datasets generated during and/or analyzed during the current study are available from the corresponding author on reasonable request.
